# Photodynamically tunable ROS-generating hydrogels for accelerated tissue regeneration

**DOI:** 10.1016/j.bioactmat.2025.05.006

**Published:** 2025-07-08

**Authors:** Seung Hee Hong, Ye Jin Park, Seo In Lee, Ki Chang Nam, Mi Hee Lee, Jong-Chul Park

**Affiliations:** a Department of Medical Engineering, Yonsei University, College of Medicine, Seoul 03722, Republic of Korea; b Graduate School of Medical Science, Brain Korea 21 Project, Yonsei University, College of Medicine, Seoul, 03722, Republic of Korea; c Department of Medical Engineering, Dongguk University, College of Medicine, Gyeonggi-do, 10326, Republic of Korea

**Keywords:** Wound healing, Hyaluronic acid, Hydrogel, Reactive oxygen species, Angiogenesis, Proliferation

## Abstract

Wound healing progresses through many key cellular activities, including fibroblast and keratinocyte proliferation and angiogenesis. This study explored the wound-healing potential of reactive oxygen species (ROS)-generating hyaluronic acid (HA) hydrogels. We fabricated a chlorin e6–conjugated HA (Ce6-HA) hydrogel that generates ROS when subjected to irradiation from an LED light source. *In vitro* studies revealed that the ROS generated by the Ce6-HA hydrogels enhanced the proliferation of fibroblasts and keratinocytes. Further, the fibroblasts were found to have high levels of intracellular ROS, elevated expression of p-ERK1/2, p-p38 MAPK, p-Akt, and cyclin D1 proteins, and enhanced collagen deposition. Moreover, the Ce6-HA hydrogel also promoted endothelial angiogenesis *in vitro*. *In vivo* studies demonstrated the ROS-generating HA hydrogels significantly improved wound closure and tissue regeneration compared to control groups. The Ce6-HA hydrogel-treated group exhibited accelerated wound healing, with enhanced fibroblast proliferation, increased keratinocyte proliferation, and better angiogenesis. Histopathological and immunohistochemical analyses showed elevated levels of key growth factors and signaling molecules, which are critical to wound healing. The controlled ROS generation from the Ce6-HA hydrogels activated broader molecular pathways necessary for effective skin tissue repair. Therefore, ROS-triggering HA hydrogels could be a viable approach to accelerate recovery and reduce scarring in clinical settings.

## Highlights

• A Ce6-conjugated HA hydrogel was developed to release ROS under red LED light for controlled wound therapy.
• ROS release promoted both fibroblast growth and keratinocyte activation, enhancing skin regeneration.
• The hydrogel stimulated angiogenesis, leading to improved vascularization and faster tissue repair.
• Histological analysis confirmed enhanced cellular activity and reduced inflammation in ROS-treated wounds.
• ROS-releasing HA hydrogels show strong therapeutic potential for wound healing by accelerating recovery.

## Graphical abstract

**Figure:**
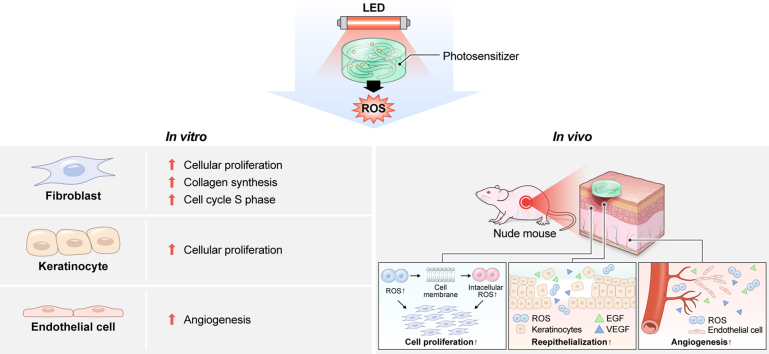
This study investigates a hyaluronic acid hydrogel conjugated with chlorin e6 (Ce6-HA), which generates ROS upon exposure to LED light. In vitro experiments were conducted using fibroblasts, keratinocytes, and endothelial cells to evaluate the cellular responses. In vivo studies demonstrated that the Ce6-HA hydrogel significantly enhanced wound closure and tissue regeneration. Histological analysis further confirmed elevated expression of growth factors and wound healing related markers.

## 1 Introduction

Wound healing is a complex and highly regulated biological process essential for restoring tissue integrity and function after injury. It progresses through a well-coordinated sequence of phases, including hemostasis, inflammation, cell proliferation, and tissue remodeling. Any disruption or delay in these stages can impair wound closure, leading to complications such as chronic wounds, excessive fibrosis, or infection, which remain major challenges in clinical practice [[1], [2], [3], [4], [5], [6]]. Among the key regulators of wound healing, reactive oxygen species (ROS) have received increasing attention due to their dual role in both promoting and potentially hindering tissue repair. While ROS are often associated with oxidative stress and cellular damage, emerging evidence suggests that they also play essential roles in modulating cell proliferation, migration, and angiogenesis when maintained within a controlled range [[7], [8], [9]].

ROS are oxygen-containing reactive molecules that are naturally produced during metabolic processes, particularly by immune cells such as neutrophils and macrophages at the site of injury [10,11]. Although excessive ROS levels can lead to oxidative damage by triggering lipid peroxidation, protein modification, and DNA damage, regulated ROS signaling is crucial for effective wound healing [12]. ROS regulate key cellular processes—including fibroblast proliferation, keratinocyte migration, and angiogenesis—which are critical for effective wound healing. Additionally, ROS influence keratinocyte proliferation and migration, which are essential for re-epithelialization and restoring the protective function of the epidermis [[13], [14], [15], [16]]. ROS-driven fibroblast and keratinocyte activation, along with VEGF-mediated angiogenesis, collectively facilitate wound closure [[17], [18], [19], [20]]. ROS signaling enhances endothelial cell proliferation and migration and upregulates vascular endothelial growth factor (VEGF), which promotes new blood vessel formation [[21], [22], [23], [24], [25]]. This angiogenic response is critical for sustaining the metabolic demands of newly formed tissue. The ability of ROS to regulate multiple aspects of wound healing has led to the development of ROS-modulating biomaterials, particularly hydrogel-based dressings, which provide a means of delivering controlled ROS levels to wound sites [25,26].

ROS modulation strategies for tissue repair have been explored using various approaches, including peroxide-loaded biomaterials, catalytic nanoparticle-integrated scaffolds, and enzyme-mimicking systems. For instance, previous studies have demonstrated that peroxide-releasing hydrogels can accelerate fibroblast proliferation, whereas nanozyme-functionalized hydrogels enable localized ROS generation to facilitate wound healing [[27], [28], [29]]. Despite these advancements, many of these systems rely on passive ROS release, which can result in inconsistent ROS levels and potential oxidative stress [[30], [31], [32]]. In contrast, the approach presented in this study introduces a novel LED-responsive, externally tunable ROS-releasing hyaluronic acid (HA) hydrogel, which allows for precise modulation of ROS generation in real-time [[33], [34], [35]]. Unlike traditional ROS-modulating materials, this system ensures that ROS levels remain within a therapeutic range, thereby avoiding excessive oxidative stress while optimizing the regenerative benefits of ROS signaling. To our knowledge, this is the first study to utilize an externally tunable Ce6-conjugated HA hydrogel for controlled ROS delivery in wound healing applications [[36], [37], [38]].

Although controlled ROS levels promote wound healing, unregulated ROS overproduction poses potential risks, including prolonged inflammation, oxidative stress-induced cellular apoptosis, and tissue fibrosis. High ROS concentrations have been shown to sustain macrophages in a pro-inflammatory M1 phenotype, which can delay wound closure and increase fibrosis risk. Additionally, excessive ROS can impair mitochondrial function, reducing ATP production and leading to cellular dysfunction [13,17,25]. To mitigate these risks, this study employs an LED-mediated ROS control system that enables precise tuning of ROS release according to the needs of the tissue microenvironment. By adjusting LED intensity and exposure time, the system prevents ROS accumulation beyond a beneficial threshold, ensuring a balance between pro-healing effects and oxidative safety.

**Scheme 1:**
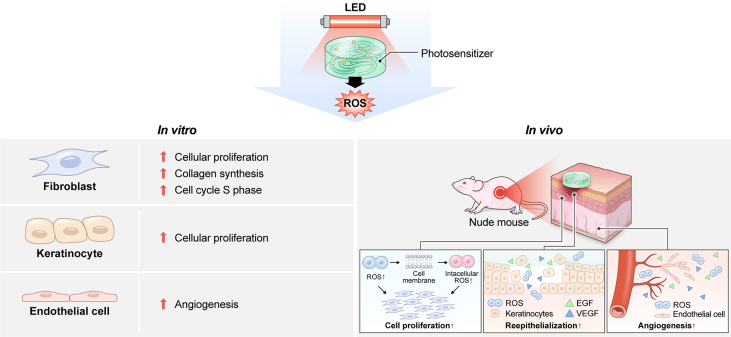
This study investigates a hyaluronic acid hydrogel conjugated with chlorin e6 (Ce6-HA), which generates ROS upon exposure to LED light. In vitro experiments were conducted using fibroblasts, keratinocytes, and endothelial cells to evaluate the cellular responses. In vivo studies demonstrated that the Ce6-HA hydrogel significantly enhanced wound closure and tissue regeneration. Histological analysis further confirmed elevated expression of growth factors and wound healing-related markers.

Building on these insights, this study focused on the development of a novel HA hydrogel capable of generating controlled levels of ROS. The primary objective was to fabricate this ROS-generating HA hydrogel and evaluate its effectiveness in enhancing wound healing both *in vitro* and *in vivo*. By confirming the beneficial effects of controlled ROS release on fibroblast proliferation, keratinocyte proliferation, and endothelial cell angiogenesis, this study seeks to establish the hydrogel as a potent tool for improving wound-healing outcomes. The findings could lead to the development of more effective wound-care products that harness the therapeutic potential of ROS while minimizing their associated risks (Scheme 1).

## 2 Materials and methods

### 2.1 Preparation of chlorin e6 (Ce6)-conjugated HA hydrogels (Ce6-HA)

Ce6-conjugated HA hydrogels were fabricated following a previously established protocol [39]. In brief, sodium hyaluronic acid (200 kDa, 200 mg, HA, Lifecore Biomedical) was dissolved in 10 ml PBS. Subsequently, 1-ethyl-3-(3-dimethylaminopropyl) carbodiimide (63.6 mg, EDC), N-hydroxysuccinimide (35.2 mg, NHS), selenocystamine (8.8 mg), and Ce6 (30 mg, Santa Cruz Biotechnology, USA) were sequentially introduced into the mixture. The resulting solution was transferred to an ultrafiltration centrifuge tube (Pall Corporation, USA) and centrifuged at 4500 rpm for 20 min at 20 °C. Following centrifugation, the crosslinker, polyethylene glycol diglycidyl ether (PEGDE), was added and the solution was transferred to a Petri dish. The Ce6-HA hydrogel was allowed to form into a circular shape with a diameter of 1 cm^2^. The hydrogel was then sterilized using 70 % ethanol and subsequently rinsed with sterile distilled water to remove any residual ethanol.

### 2.2 Light-emitting diode (LED) power controller

A custom-designed red LED system was employed as the light source for photodynamic therapy (PDT) control. The LED system was configured to emit light with a peak wavelength of 660 nm. Additionally, the system was equipped with filters to eliminate the majority of ultraviolet (UV) radiation. The distance between the LED source, and the sample was maintained at over 5 cm. The irradiance of the light source was precisely measured using a power meter (S130C, Thorlab, Inc., USA) immediately prior to the experiment.

### 2.3 Estimation ROS released by irradiation of Ce6-HA hydrogels

The photocatalytic activity of the Ce6-HA hydrogel was evaluated using 1,3-diphenylisobenzofuran (DPBF; Sigma Aldrich) as an ROS indicator. Ce6-HA samples were immersed in a DPBF solution and subsequently exposed to red LED at irradiance levels of 50, 100, 250, 500, 750 μW/cm^2^, and 1 mW/cm^2^. The total exposure duration was 40 min, with readings taken at 10 min intervals. Following irradiation, the DPBF solution was transferred to a quartz cuvette, and its UV–Vis absorbance spectrum was measured using a spectrophotometer at 411 nm (Shimadzu, Kyoto, Japan).

### 2.4 Effect of ROS on cell proliferation

Neonatal human dermal fibroblasts (NHDFs, Lonza, Switzerland), a type of fibroblast cell, were cultured in fibroblast growth medium (Lonza) and culture media was replaced with fresh medium every 3–4 days. NHDFs were seeded into a 48 well plate at a density of 1 × 10^4^ cells/cm^2^ and incubated overnight at 37 °C in a CO_2_ incubator for cell attachment. HaCaT (ATCC®) cells were used as a representative keratinocyte model cultured in Dulbecco’s Modified Eagle’s Medium containing 10 % fetal bovine serum and 1 % antibiotics (Welgene, Seoul, Korea). The HaCaT cells were seeded at a density of 10,000 cells/cm^2^ in a 48 well plate and incubated overnight at 37 °C in a CO_2_ incubator to ensure proper cell attachment. Following incubation, each well was treated with either HA or Ce6-HA, and the plates were subsequently exposed to varying intensities of red LED for different durations. Cell viability was assessed using the Cell Counting Kit-8 (CCK-8) assay at 4, 24, and 72 h post-irradiation. After the specified exposure times, CCK-8 solution was added to each well, and the plates were incubated for 2 h in the dark to prevent light interference. The absorbance was then measured at 450 nm using a spectrophotometer. The growth rate was presented as a comparison to the control group, which measured cell viability 4 h after HA treatment.

### 2.5 Intracellular ROS detection

Intracellular ROS levels were quantified using the Oxiselect™ Intracellular ROS Assay kit. NHDFs and HaCaT cells were seeded at a density of 1 × 10^4^ cells/cm^2^ in a well plate and incubated overnight to allow cell attachment. Following incubation, the cells were washed with PBS and treated with a 1x DCFH-HA solution in the culture medium, followed by a 1 h incubation at 37 °C. After washing the cells again with PBS, the Ce6-HA hydrogel treatment was applied, followed by LED irradiation at an irradiance of 100 μW/cm^2^ for varying durations, followed by a 1 h incubation period. The cells were then rinsed with PBS, and a cell lysis buffer was added to each well. The fluorescence of the oxidized product, 2′,7′-dichlorodihydrofluorescein, was measured using a microplate reader set to an excitation wavelength of 480 nm and an emission wavelength of 530 nm.

### 2.6 Effect of ROS on fibroblast

#### 2.6.1 Cell cycle analysis

NHDFs were seeded at a density of 1 × 10^4^ cells/cm^2^. The following day, the cells were treated with either HA or Ce6-HA, followed by LED irradiation at 100 μW/cm^2^ for 30 min. After 24 and 48 h, the cells were harvested by detachment from the well plate using trypsin-EDTA. The collected cells were then washed twice with ice-cold PBS and centrifuged at 1200 rpm for 5 min at 4 °C. The resulting cell pellet was treated with 70 % ethanol and incubated at 4 °C for 30 min. After a subsequent centrifugation at 1200 rpm for 5 min at 4 °C, the ethanol supernatant was carefully removed. RNase was then added to the cell pellet, and the mixture was incubated for 30 min at room temperature. Propidium iodide (PI) solution was subsequently added to the cells and incubated for 30 min in the dark at ambient temperature. Following staining, the samples were analyzed using a flow cytometer (BD FACSymphony A5).

#### 2.6.2 Western blot

NHDFs were seeded in a well plate, incubated overnight, and subsequently treated with either HA or Ce6-HA. Following LED irradiation at 100 μW/cm^2^ for 30 min, the cells were harvested at 24, 48, and 72 h using a protein lysis buffer. Protein concentrations were determined using the Bradford assay. Equal amounts of protein samples were then loaded onto a 12.5 % SDS-PAGE gel and subjected to electrophoresis at a constant voltage until sufficient protein separation was achieved. The separated proteins were transferred onto a polyvinylidene difluoride membrane. To prevent non-specific antibody binding, the membrane was blocked with 5 % skim milk. Subsequently, the membrane was incubated overnight at 4 °C with gentle agitation in a 5 % BSA solution containing primary antibodies specific to p-ERK1/2, EKR1/2, p-p38 MAPK, p38 MAPK, p-AKT, AKT, cyclin D1, and β-actin (Cell Signaling Technology, Danvers, MA, USA). After washing away unbound primary antibodies with TBST buffer, the membrane was incubated with horseradish peroxidase-linked anti-rabbit or anti-mouse IgG secondary antibody (Cell Signaling Technology) for 1 h at RT. Following a final TBST wash to remove any unbound secondary antibodies, the protein bands were visualized using a chemiluminescent substrate (SignalFire ECL reagent, Cell Signaling Technology).

#### 2.6.3 Collagen assay

Collagen levels were assessed using the Sircol Soluble Collagen Assay kit (Biocolor Assays, United Kingdom) according to the manufacturer’s instructions. Briefly, collagen deposition was quantified using a dye reagent specific to collagen. NHDFs treated with HA or Ce6-HA exposed to LED 100 μW/cm^2^ for 30 min or 60 min were fixed in 4 % paraformaldehyde and subsequently stained with Sirius Red solution for 30 min at RT. After the staining procedure, the samples underwent washing with acid-salt wash reagent, followed by the addition of 0.1M NaOH to elute the bound dye, which was allowed to react for 5 min. Absorbance was measured at 556 nm using a microplate reader to quantify the collagen. To determine the total protein content, a bicinchoninic acid (BCA) assay was performed. Samples were treated with BCA working solution and incubated at 37 °C for 30 min. Absorbance was then measured at 562 nm using a plate reader. A standard curve was generated using bovine serum albumin (BSA) as the standard.

### 2.7 Angiogenesis assay

HUVECs were seeded onto Matrigel-coated well plates at a density of 3 × 10^4^ cells/cm^2^. Following seeding, 0.2-μm microporous membrane transwells were introduced, and the cells were treated with either HA or Ce6-HA. HUVECs were categorized into several groups: a control group with no intervention, a group receiving HA treatment alone, a group treated with a combination of HA and VEGF as a positive control, a group treated with HA followed by LED exposure, and a group treated with Ce6-HA followed by LED exposure. LED irradiation was set at 100 μW/cm^2^ for 30 min. Following cell seeding and treatment application, live/dead assays were performed using Endothelial Tube Formation Assay kit (Cell Biolabs, San Diego, USA), following manufacturer’s instructions, after 6 h to evaluate the length of cell connections and the number of junctions formed. VEGF treatment served as the positive control. Tube formation was subsequently observed using a microscope, and quantitative analysis was performed using ImageJ software. The parameters evaluated included the number of junctions and the total tube length.

### 2.8 *In vivo* studies

#### 2.8.1 Animal experiment approval

An animal study was conducted in accordance with the guidelines set forth in the “Guide for the Care and Use of Laboratory Animals.” The study protocols were approved by the Institutional Animal Care and Use Committee (IACUC) of the Yonsei Laboratory Animal Research Center (YLARC; Permit Number: 2023-0136). The animals were housed in a pathogen-free environment at YLARC. Five-week-old male Balb/c nude mice, procured from Orient Bio, were used to establish a full-thickness wound model. Prior to the experiment, the Balb/c nude mice underwent a 1-week acclimation period, and the mice were then randomly assigned to the experimental groups.

#### 2.8.2 Surgery for the creation of full-thickness wounds

**Fig. 1:**
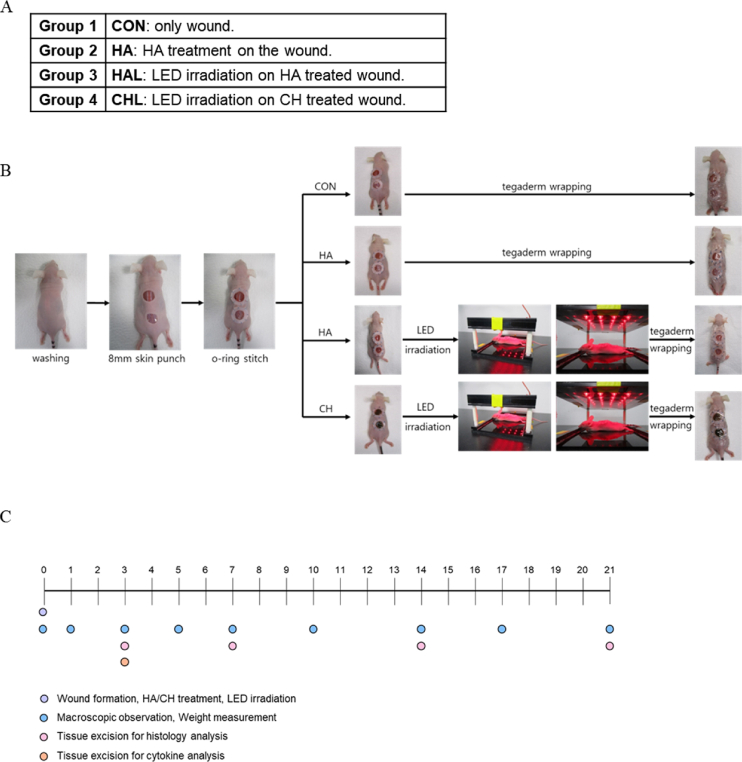
Animal experiment group classification and schedule (A) The animals were divided into four groups; only wound (CON), HA treatment on the wound (HA), LED irradiation on HA-treated wound (HAL), and LED irradiation on Ce6-HA treated wound (CHL). (B) ROS-induced wound healing model of balb/c nude mouse. (C) Every 3–4 d, macroscopic examination was carried out to assess the size of the wound and its body weights. Every week, tissue was excision for histology analysis.

Four distinct groups were created for the *in vivo* investigation: control (CON), wound treated with HA (HA), wound treated with HA combined with LED irradiation (HAL), and wound treated with Ce6-HA combined with LED irradiation (CHL). Mice were anesthetized via intraperitoneal injection of zoletil (35 mg/kg) and rompun (2 mg/kg). The surgical site was sterilized with a 70 % ethanol solution. While under anesthesia, two full-thickness skin wounds, each 8 mm in diameter, were created on the dorsal surface of the mice using a biopsy punch. A round silicone splint was positioned around the wound and secured to the skin with 4-0 nylon sutures to maintain its placement. The hydrogel was positioned after the silicone splint was sutured. The group designated for LED treatment was exposed to LED at an intensity of 200 μW/cm^2^ for 30 min. A preliminary experiment on LED irradiance showed that 200 μW/cm^2^ for 30 min was the most effective condition for wound contraction and angiogenesis promotion, and these parameters were subsequently used in the current study (Supplementary 1). Following each procedure, the wound site was protected with an occlusive polyurethane dressing (Tegaderm™, 3M). Macroscopic assessment of wound healing was performed by photographing the wound using a digital camera and recording the weight of the mice for 3–4 days interval. Photographs were taken to monitor the healing process and assess changes in wound area over time. Upon completion of the study, the animals were euthanized using CO_2_ chamber, and skin tissue samples were collected from the center of each wound for further analysis (Fig. 1A–C).

#### 2.8.3 Hispathological examination

The injured skin tissue was collected and promptly fixed in 10 % paraformaldehyde for 24 h. Following fixation, the tissue samples were embedded in paraffin and sectioned into 5 μm thick slices. The sections were stained using hematoxylin and eosin (H&E) and Masson’s Trichrome (MT) staining techniques. The stained slides were then examined under a brightfield microscope (Olympus BX51; Olympus, Tokyo, Japan) to assess the histological features.

#### 2.8.4 Immunohistological analysis

To assess angiogenesis, fibroblast formation and macrophages, the tissue sections were immunohistochemically stained for PCNA, CD31, vimentin and CD68. PCNA was studied in day 7 tissue samples, while both CD31 and PCNA were studied in day 14 tissue samples. CD68 was studied in day 3, 7, 14, 21 tissue sample. The skin tissue samples were embedded in paraffin and sectioned into 5-μm-thick slices. The sections were incubated in Tris/EDTA buffer (pH 9.0) at room temperature, followed by overnight incubation with primary antibodies. Then, the slides were rinsed with PBS and subsequently incubated with secondary antibodies for 2 h at RT. The stained samples were examined under a light microscope, and signal intensities were quantified using ImageJ software. All experiments were performed in triplicate.

#### 2.8.5 Analysis of cytokine extracted from mouse skin tissue

Skin samples of uniform size were collected from the animals on day 3 after wound creation and homogenized in PBS. A skin sample of 1 cm^2^ was obtained from the central area of the wound. The resulting tissue homogenates were utilized for subsequent analyses. The concentrations of epidermal growth factor (EGF) and VEGF were quantified using enzyme-linked immunosorbent assay (ELISA) kits from R&D Systems (London, UK). The assays were performed according to the manufacturer’s instructions. Cytokine levels were calculated using standard curves, and the results are expressed as the amount of cytokines detected.

### 2.9 Statistical analysis

Statistical analysis was conducted using SPSS 26.0. One-way ANOVA followed by Tukey’s post hoc test was used for multiple group comparisons. Student’s t-test was used for pairwise comparisons. A p-value of <0.05 was considered statistically significant. Data are presented as mean ± standard deviation (SD).

## 3 Results and discussion

### 3.1 ROS generation

**Fig. 2:**
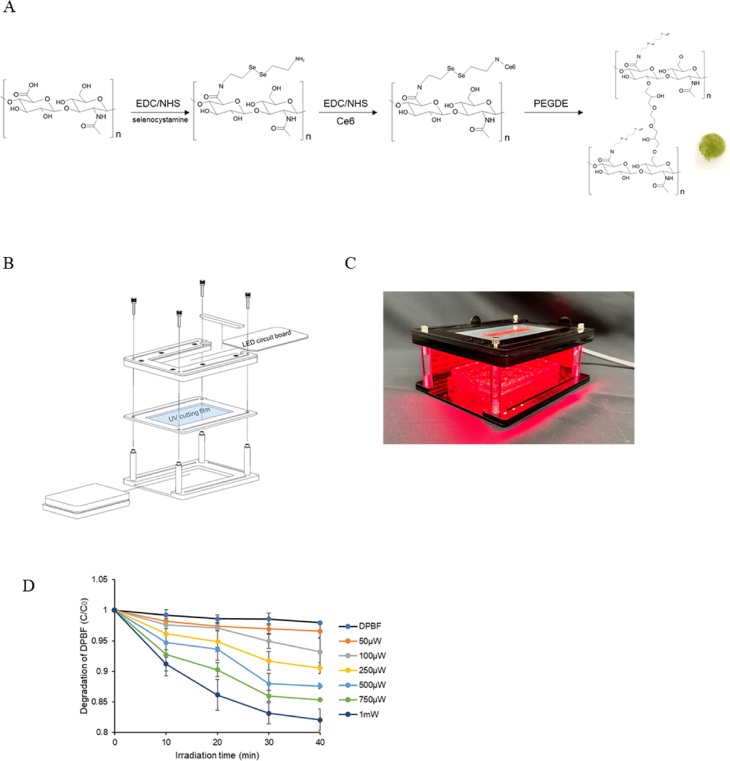
Preparation of Ce6-HA hydrogels, LED irradiation device setup, and ROS detection (A) Schematic illustration of the preparation of crosslinked Ce6-bonded HA hydrogel. (B) Blue print of LED equipment. (C) Photograph of the LED equipment emitting light. (D) Degradation of DPBF measured upon LED irradiation at 50, 100, 250, 500, 750 μW/cm^2^ and 1 mW/cm^2^ at 10 min intervals. Data are presented as mean ± SD (n = 5).

The preparation scheme of the Ce6-HA hydrogel is shown in Fig. 2A. LED irradiation–induced ROS production by the Ce6-HA hydrogel was quantified by monitoring the decomposition of DPBF, an ROS indicator. In the presence of ROS, the yellow DPBF is converted into a colorless compound [[36], [37], [38]]. The ROS generated by LED-irradiated Ce6-HA was assessed by varying both the LED power and irradiation time. Irradiation was conducted using red LED at different intensities, with measurements taken every 10 min. The blue print of the LED equipment and a photograph of the LED emitting light are shown in Fig. 2B and C. Power was always measured before performing the experiment, and ice was placed on top to prevent heat generation. C_0_ represents the DPBF solution without any irradiation. To ensure comparability across conditions, ROS generation was normalized to baseline DPBF absorbance (C_0_) and expressed as a ratio (C/C_0_) relative to non-irradiated controls. Ce6 was successfully conjugated to HA, and as a result, it was observed that the generation of ROS was proportional to the intensity and duration of light exposure (Fig. 2D). Peptide bonds were successfully formed in hyaluronic acid through EDC/NHS coupling, enabling the conjugation of Ce6 to the hyaluronic acid. Chemical modifications can facilitate the formation of amide bonds with HA, allowing for the attachment of various functional groups, peptides, or pharmaceuticals. This process broadens the applicability of HA in drug delivery, tissue engineering, and other biomedical areas. The modification involves activating the carboxyl groups of HA using agents like carbodiimides or N-hydroxysuccinimide, which then react with primary amines to create stable amide bonds [34]. This amidation process not only alters the physical, chemical, and biological properties of HA, but also transforms it into a versatile biomaterial with customizable characteristics for specific uses [40]. Thus, HA molecules are modified to incorporate functional groups that can form amide bonds with reactive sites on a photodynamic agent.

### 3.2 Fibroblast proliferation enhancement via ROS generation from Ce6-HA hydrogels

**Fig. 3:**
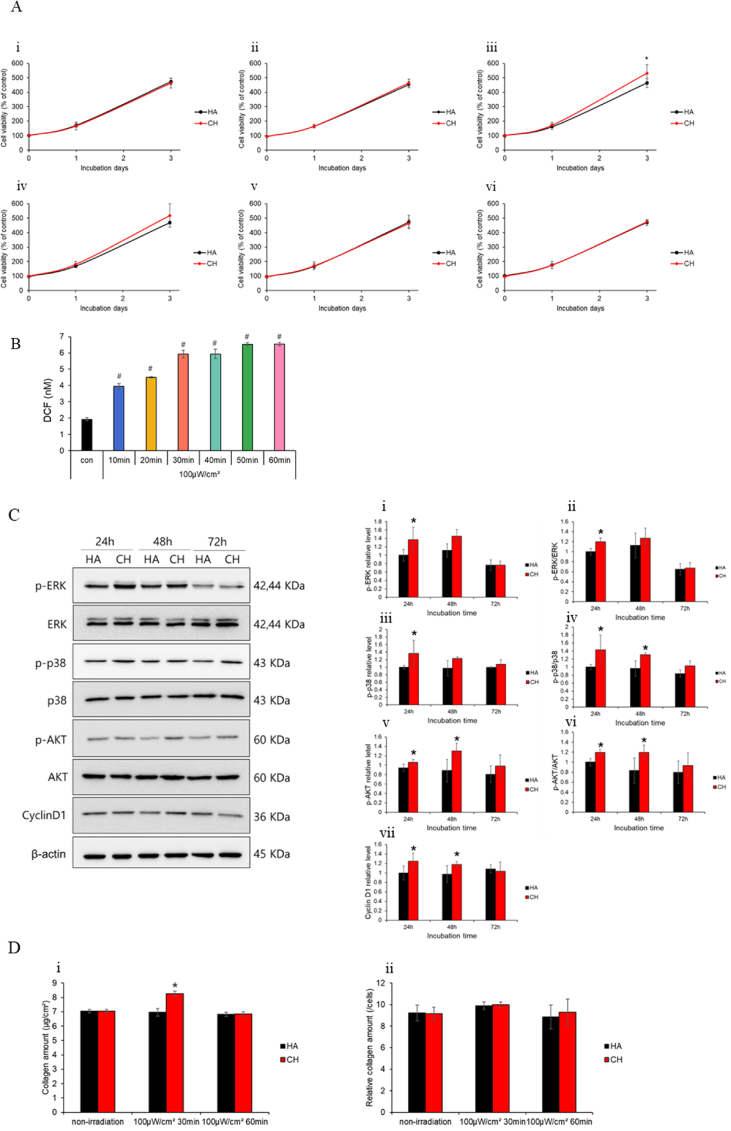
Assessment of fibroblast response to ROS-induced effects (A) Cellular proliferation assay using NHDF cells subjected to LED irradiation (100 μW/cm^2^) for different durations. (i) Non-irradiation, (ii) 20 min, (iii) 30 min, (iv) 40 min, (v) 50 min, (vi) 60 min LED irradiation. ∗p < 0.05 vs. HA (n = 5). (B) Estimated intracellular ROS levels at different irradiation time points under 100 μW/cm^2^ intensity. #p < 0.01 vs. con (n = 3). (C) Cells were harvested after 24, 48, and 72 h after LED irradiation. p-ERK1/2, ERK1/2, p-p38 MAPK, p38 MAPK, p-AKT, AKT, cyclin D1 and β-actin proteins levels were measured using Western blot. The graphs show quantified proteins levels; (i) relative p-ERK1/2 level, (ii) p-ERK1/2/ERK1/2, (iii) relative p-p38 MAPK level, (iv) p-p38 MAPK/p38 MAPK, (v) relative p-AKT level, (vi) p-AKT/AKT (vii) relative cyclin D1 level. Data are expressed as means ± SD (n = 3). ∗p < 0.05 vs. 24 h HA (D) (i) Total collagen estimated using the Sircol soluble collagen assay kit. (ii) Collagen amount normalized to total protein content showing increased collagen level due to the higher fibroblast number. ∗p < 0.05 vs. HA (n = 3). Throughout all figures and graphs in the *in vitro* experiments, HA represents the group using HA hydrogel, while CH denotes the group using Ce6-HA hydrogel. This labeling is consistently applied across all in the *in vitro* data representations in the manuscript.

Following overnight seeding of NHDF cells, the cells were treated with either HA or Ce6-HA hydrogels. Subsequently, the cells were exposed to 100 μW/cm^2^ LED for varying durations to induce ROS generation. Cell viability was evaluated at 4, 24 and 72 h post-irradiation, as well as on the day of light exposure. In the control group that was not irradiated, no significant difference in cell growth was observed between the HA-treated and Ce6-HA-treated groups. However, in the group treated with Ce6-HA and irradiated for 30 min, a 15 % increase in cell growth was observed on the third day compared to the group treated with HA followed by irradiation. HA treated group exhibited similar growth regardless of LED irradiation, while Ce6-HA treated group showed a slight increase in growth at 40 min, though the difference was not statistically significant. Notably, variations in the duration of irradiation, whether shorter or longer than 30 min, did not influence cell growth (Fig. 3A). It can be confirmed that an appropriate amount of ROS positively influences cell growth promotion. A 15 % increase in fibroblast proliferation could significantly enhance wound healing, particularly in the context of accelerated tissue repair and reduced healing time. The increased fibroblast proliferation observed in response to ROS contributes to ECM remodeling and collagen synthesis [41]. This enhancement might also contribute to faster wound contraction and a quicker transition from the inflammatory to the proliferative phase of healing, potentially reducing the risk of complications like chronic wounds or excessive scarring [42]. Moreover, the increased fibroblast activity would likely lead to a higher production of growth factors and cytokines, which are crucial for coordinating the overall healing process, including the activities of other key cells, such as keratinocytes and endothelial cells [43]. Thus, even a modest improvement in fibroblast proliferation could have a meaningful impact on the efficiency and quality of wound healing, particularly in clinical settings, where faster recovery is desired.

### 3.3 Effect of LED irradiation of Ce6-HA hydrogels on intracellular ROS levels

ROS function as signaling molecules that activate various pathways involved in cell proliferation. These pathways regulate cell cycle progression and enhance cell proliferation. Consequently, it can be inferred that appropriate light exposure for an optimal duration may lead to the generation of ROS, which could positively impact cellular viability in NHDF cells [44,45]. The oxidized DCF fluorescent marker was employed to monitor the generation of intracellular ROS. A significant increase in relative fluorescence units was observed following irradiation at 100 μW/cm^2^. These findings indicate that the generation of extracellular ROS can stimulate the production of intracellular ROS. The ROS concentration generated by the interaction of light with Ce6-HA for 30 min effectively stimulated fibroblast proliferation (Fig. 3B). It was confirmed that irradiation at 100 μW/cm^2^ for 30 min significantly promoted cell growth. Intracellular ROS levels were similar between the 30 min and 40 min LED irradiation, but no significant cellular proliferation results were observed for the 40 min irradiation. While there was no significant difference in intracellular ROS between the 30 min and 40 min irradiation, significant differences were observed at 10, 20, 50, and 60 min. LED-induced ROS generation leads to intracellular ROS accumulation, which activates downstream proliferation pathways [[46], [47], [48]]. These connections are crucial for understanding the broader impact of ROS in cellular physiology and pathology, particularly in contexts like wound healing, where both extracellular and intracellular ROS play significant roles.

### 3.4 Effect of ROS generation on the cell cycle and cellular pathway

Cell cycle analysis is a widely utilized technique that enables researchers to investigate and categorize the various stages of a cell’s life cycle, encompassing growth, DNA replication, and division. This technique typically delineates the cell cycle into four distinct phases: G1, S, G2, and M. In the G1 phase, cells prepare for DNA replication, which occurs during the subsequent S phase. The G2 phase involves further cellular growth and preparation for mitosis, while the M phase is characterized by the division of the cell nucleus [[49], [50], [51], [52], [53]]. An increase in the number of cells in the S phase is indicative of heightened cellular proliferation, as a greater number of cells undergo division and enter the S phase for DNA replication [46]. After hydrogel treatment and LED irradiation followed by a 48-h incubation, cell cycle analysis revealed an elevation in the S phase, signifying enhanced cellular proliferation. Both the control group and the LED-only group exhibited similar cell cycle patterns. However, when LED irradiation was combined with HA and Ce6-HA treatment, followed by a 48 h incubation, a marked increase in the S phase was observed, particularly in the Ce6-HA-treated group. This suggests that ROS generated by Ce6-HA influence the NHDF cell cycle, thereby promoting cell proliferation (Table 1). HA plays a crucial role in various physiological processes such as tissue regeneration, wound healing, cell migration, and cell proliferation. HA mainly functions extracellularly by interacting with cell receptors, indirectly supporting cell proliferation. While CD44, a cell receptor, can activate signals for cell migration and proliferation when bound to HA, whether this leads directly to S phase entry depends on cell type and experimental conditions [[33], [34], [35]]. At controlled levels, ROS act as signaling molecules that promote cell cycle progression. They do this by activating various signaling pathways, such as the mitogen-activated protein kinase (MAPK) pathway, which is crucial for the transition from the G1 to the S phase of the cell cycle. ROS can also modulate the activity of cyclin-dependent kinases (CDKs) and cyclins, proteins that directly control the progression through different phases of the cell cycle [53].

**Table 1:** Analysis of fibroblast cell cycle in response to ROS.

	Cell cycle phase	Non irradiation	LED irradiation	HA	CH + LED irradiation
24 h	G_0_/G_1_	62.8 %	62.1 %	54.1 %	49.7 %
	S	21 %	21.8 %	29.9 %	30 %
	G_2_	16 %	15.8 %	15.5 %	20.1 %
48 h	G_0_/G_1_	75.1 %	74.6 %	58.7 %	54.1 %
	S	12.6 %	12.3 %	22.5 %	28.3 %
	G_2_	11.9 %	12.7 %	18.7 %	17.1 %

Cellular proliferation is regulated by numerous signaling pathways, many of which are influenced by ROS production. Notably, the ERK1/2, p38-MAPK, and AKT pathways are among those affected by ROS. ROS have a significant influence on the ERK1/2, p38-MAPK, and AKT signaling pathways, each contributing to the regulation of cell fate. ERK1/2 and AKT pathways generally promote cell survival and proliferation under moderate ROS levels. The p38-MAPK pathway is associated with stress responses, leading to cell cycle arrest or apoptosis under high ROS conditions. The balance of the activation of these pathways is crucial for maintaining cellular homeostasis and preventing diseases, such as cancer [[54], [55], [56], [57], [58], [59], [60], [61], [62]]. We investigated these pathways in NHDF cells treated with HA or Ce6-HA, collecting samples at 24, 48, and 72 h after LED irradiation. We observed that the expression levels of p-ERK1/2, p-p38-MAPK, and p-AKT proteins were elevated in cells treated with Ce6-HA compared with those treated with HA at both 24 and 48 h post-exposure with β-actin used as the housekeeping protein, correlating with enhanced proliferative signaling. However, by the 72 h mark, the expression of proteins associated with cell proliferation declined in both groups, likely owing to the cells reaching confluence in the well plate. Additionally, proteins involved in the S phase of the cell cycle, such as cyclin D1, exhibited increased expression levels in Ce6-HA treated cells relative to HA-treated cells. To quantify these changes, all protein expression levels were normalized to β-actin as a housekeeping control, and relative phosphorylation levels (p-ERK1/2/ERK1/2, p-p38/p38, p-AKT/AKT) were standardized by setting the 24 h HA condition to 1.0. Notably, the p-ERK1/2/ERK1/2 and p-AKT/AKT ratios peaked at 24 h and remained elevated at 48 h, supporting their role in driving cell cycle progression. However, by 72 h, phosphorylation levels of all three proteins declined, likely due to confluence-related contact inhibition, resulting in reduced proliferative signaling. This trend suggests that ROS-mediated activation of ERK1/2 and AKT promotes NHDF proliferation in a time-dependent manner, with sustained signaling up to 48 h post-treatment (Fig. 3C). ROS contribute to the regulation of the cell cycle by increasing cyclin D levels, which is essential for cell proliferation. This effect is mediated through the activation of key signaling pathways, such as ERK1/2 and AKT, which are integral to the proper functioning of the cell cycle and are particularly important in processes like wound healing where rapid cell proliferation is required [42,53].

### 3.5 Effect of ROS on collagen deposition

It is well established that fibroblasts are capable of producing collagen. Therefore, collagen production of fibroblasts was analyzed [[15], [16], [17], [18]]. As the concentration of fibroblasts increased in response to ROS, the amount of collagen produced also increased (Fig. 3D). The condition under which NHDF growth is increased is when LED irradiation is applied at 100 μW/cm^2^ for 30 min with Ce6-HA. LED irradiation for 60 min at 100 μW/cm^2^ does not affect growth. Therefore, increased collagen production due to cell growth can only be observed under the condition of 30 min of LED irradiation, where cell growth is enhanced. By normalizing the total collagen production to the total protein content, it was inferred that the observed increase in collagen production was primarily owing to an increase in cell number, indicating that collagen secretion per cell remained constant (Fig. 3D). At controlled levels, ROS function as signaling molecules that activate key pathways, such as ERK1/2, AKT, and p38-MAPK, which regulate gene expression crucial for cell cycle progression and proliferation. This results in the upregulation of cyclin D, facilitating the advancement of fibroblasts through the cell cycle, thereby increasing their proliferation. ROS-mediated fibroblast proliferation enhances collagen synthesis, which strengthens tissue structure. Furthermore, ROS not only stimulate fibroblast proliferation but also directly enhance the expression of genes involved in collagen synthesis, further promoting collagen deposition during the wound healing process [15]. However, it is essential to maintain ROS levels within a controlled range, as excessive ROS can induce oxidative stress, potentially disrupting the healing process and leading to tissue damage [42]. Thus, the dual role of ROS in fibroblast proliferation and collagen synthesis underscores their importance in effective wound repair and tissue regeneration [44,53].

### 3.6 Influence of ROS on keratinocyte proliferation

**Fig. 4:**
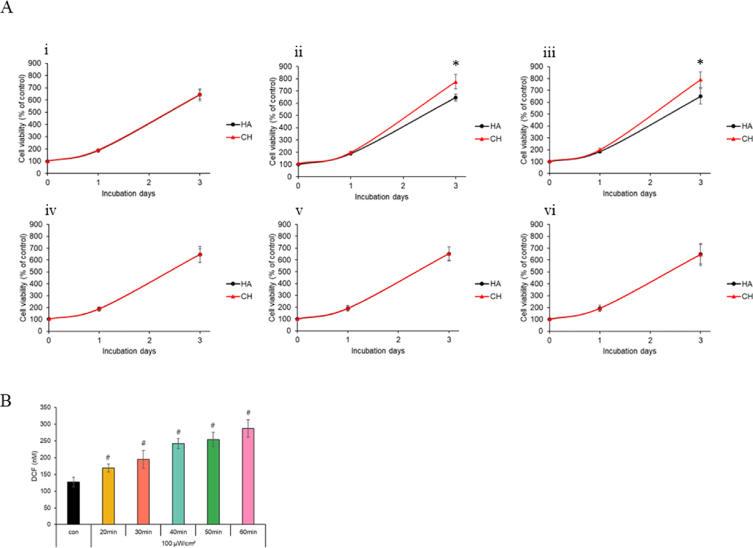
Evaluation of the effects of ROS on keratinocytes (A) HaCaT cells were treated with HA or Ce6-HA (CH) under 100 μW/cm^2^ LED irradiation for 20–60 min; (i) non-irradiation, (ii) 20 min, (iii) 30 min, (iv) 40 min, (v) 50 min, (vi) 60 min ∗ p < 0.05 vs. HA (n = 5). (B) Intracellular ROS levels induced in keratinocytes upon Ce6-HA treatment and LED irradiation. #p < 0.01 vs. con (n = 3).

The epidermis, the outermost layer of the skin, is primarily composed of keratinocytes. The proliferation of these cells is essential for maintaining skin integrity and function. During wound healing, keratinocytes play a critical role in re-epithelialization, wherein they migrate and proliferate to cover the wound site, thereby restoring skin integrity and preventing infection [[63], [64], [65], [66], [67], [68]]. At physiological levels, ROS act as signaling molecules that enhance keratinocyte proliferation. ROS signaling promotes keratinocyte proliferation through mechanisms similar to fibroblast activation. These pathways facilitate the progression of keratinocytes through the cell cycle, promoting their proliferation and contributing to the re-epithelialization of wounds [69]. During wound healing, ROS levels increase transiently, which is essential for keratinocyte activation. These activated keratinocytes proliferate and migrate to cover the wound bed, restoring the epidermal barrier [70]. HaCaT cells did not exhibit any differences in growth in the absence of LED irradiation. However, when exposed to 100 μW/cm^2^ of LED for 20 or 30 min, Ce6-HA treatment resulted in a significant 20 % increase in cell growth after 3 d compared to HA treatment with LED irradiation. Notably, LED irradiation for more than 40 min did not result in any proliferation differences in cell growth (Fig. 4A).

We investigated intracellular ROS levels by exposing cells to LED irradiation at an intensity of 100 μW/cm^2^ and measuring ROS at 10 min intervals. Our observations demonstrated a proportional increase in ROS levels over the study period, suggesting that an optimal amount of ROS stimulates intracellular processes. This elevation in ROS activity may have contributed to enhanced cell proliferation (Fig. 4B). These findings provide valuable insights into the physiological responses of cells and offer new perspectives on cell growth and metabolic functions. This study could serve as a foundation for future advancements in therapeutic treatments and the development of regulatory mechanisms.

The differential proliferation behaviors of fibroblasts and keratinocytes under similar ROS levels can be attributed to their distinct redox sensitivities and intracellular antioxidant capacities. Previous studies have demonstrated that fibroblasts exhibit higher oxidative stress resistance due to upregulated antioxidant defenses, whereas keratinocytes are more susceptible to ROS-induced stress responses [[71], [72], [73], [74]]. Additionally, fibroblasts predominantly activate ERK/Akt signaling to promote ECM remodeling under moderate ROS conditions, while keratinocytes rely on p38/JNK stress pathways, which can induce growth arrest at elevated ROS levels [[75], [76], [77]].

This highlights the necessity of fine-tuning ROS modulation to achieve optimal cellular responses for wound healing applications. While ROS generation is generally proportional to light intensity and irradiation duration, several biological and physicochemical mechanisms regulate ROS levels, preventing excessive accumulation. Oxygen depletion naturally limits ROS production, while Ce6 photobleaching under prolonged irradiation reduces its ROS-generating efficiency. Additionally, cellular antioxidant defenses such as NRF2-mediated activation of SOD, catalase, and glutathione peroxidase and neutralize excess ROS, and negative feedback mechanisms through MAPK/p38 and NF-κB signaling mitigate oxidative stress. ROS diffusion and clearance dynamics further restrict excessive accumulation. In this study, despite prolonged irradiation, ROS levels remained within a physiologically tolerable range, suggesting a well-regulated oxidative environment conducive to wound healing.

### 3.7 Enhancement of endothelial cell angiogenesis through ROS

**Fig. 5:**
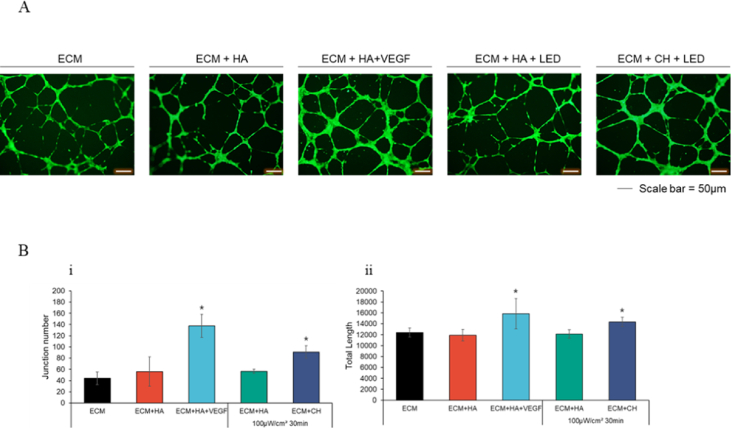
Effect of ROS on tube formation analyzed using fluorescence staining (A) Confirmation of angiogenesis using the tube formation assay. Fluorescence image of tube formation. (B) (i) The junction number and (ii) total tube length was analyzed using image J. ∗p < 0.05 vs. ECM + HA (n = 5).

Under LED exposure conditions optimized for promoting fibroblast and keratinocyte growth, we evaluated the effects of LED exposure on HUVECs to assess their potential for proliferation. However, no significant effect on HUVEC proliferation was observed under these conditions. In contrast to NHDF and HaCaT cells, which exhibited increased proliferation, HUVECs did not show any signs of proliferation or apoptosis. When the same conditions were applied to assess tube formation, an increase was observed. The control, HA-treated, and HA-treated groups with subsequent LED exposure showed similar results. In contrast, the groups treated with HA and VEGF or Ce6-HA followed by LED exposure exhibited significantly higher values. This suggests that LED exposure induced ROS generation in HUVECs treated with Ce6-HA, which significantly enhanced angiogenesis, although not to the same extent as VEGF (Fig. 5A,B). ROS play a significant role in promoting angiogenesis, particularly in HUVECs, which are widely used as a model to study vascular biology [78]. ROS enhance endothelial cell angiogenesis via VEGF signaling [79]. VEGF, a key angiogenic factor, induces ROS production in endothelial cells, which further amplifies angiogenesis by promoting cell proliferation, migration, and the expression of pro-angiogenic genes [80]. Additionally, ROS enhance HUVEC migration and the formation of capillary-like structures, critical steps in angiogenesis. This is often mediated by the activation of NADPH oxidase, a primary source of ROS in endothelial cells, which modulates pathways involved in cytoskeletal rearrangement and cell adhesion [81].

### 3.8 Wound healing potential of ROS-generating hydrogels

**Fig. 6:**
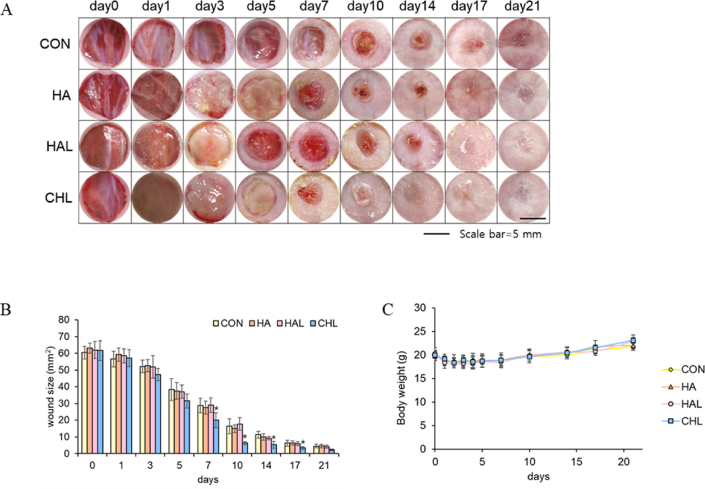
Wound healing assessment *in vivo* (A) Representative images of wound healing progression in Balb/c mice. Full-thickness wounds were created using a biopsy punch, and wound size was measured from photographs taken on days 0, 1, 3, 5, 7, 14, 17, and 21. Scale bar = 5 mm. (B) Wound area reduction analysis. Wound sizes were quantified from day 0 to day 21, and statistical comparisons were made. ∗p < 0.05 vs. HA (n = 3). (C) Body weight monitoring of Balb/c nude mice during the observation period (n = 3).

Wound healing is a complex physiological process that involves a range of cellular and molecular mechanisms aimed at restoring tissue structure and function. *In vivo* experimental studies on skin wounds are crucial for evaluating the efficacy of various therapeutic interventions in promoting wound closure and tissue regeneration. In this study, four different treatment models were employed to assess wound healing potential. The results demonstrated that the CHL treatment produced the most significant reduction in wound size by the seventh day post wound creation, suggesting a synergistic effect between Ce6-HA and LED irradiation (Fig. 6A,B). While this study successfully demonstrates effective ROS generation and biocompatibility within the tested conditions, we acknowledge certain limitations related to long-term ROS stability and hydrogel biocompatibility. Prolonged ROS generation may be influenced by Ce6 photobleaching and oxygen depletion, potentially reducing ROS efficiency over extended periods. To maintain therapeutic efficacy over longer durations, future investigations could explore modulating irradiation patterns or incorporating oxygen-releasing biomaterials to enhance ROS stability. Additionally, while short-term biocompatibility has been confirmed, the long-term effects of hydrogel degradation and chronic ROS exposure require further study. Future work will focus on evaluating hydrogel degradation kinetics, oxidative stress responses, and potential systemic effects through extended *in vivo* studies to optimize long-term safety and therapeutic performance [82,83]. Animal body weight showed a temporary decrease following surgery; however, no significant differences were observed among experimental groups. These findings suggest that CHL does not induce adverse systemic effects, providing evidence to support its biocompatibility and safety (Fig. 6C). This study aimed to investigate the underlying mechanisms contributing to the accelerated wound healing observed with the combination of Ce6-HA and LED irradiation therapy. The combined use of Ce6-HA and LED irradiation in wound healing underscores the potential of ROS induction as a therapeutic strategy to enhance tissue regeneration and wound closure. This combination appears to amplify the physiological processes critical for wound repair, including collagen synthesis, angiogenesis, and epithelialization. ROS play a critical role in various cellular processes integral to wound healing, including the resolution of inflammation, collagen synthesis, angiogenesis, and epithelialization [54]. By leveraging the ability of Ce6-HA to induce ROS production, we can amplify the physiological mechanisms that facilitate wound repair and expedite the healing process. Our findings suggest that the light conditions used generate minimal heat, thereby eliminating any detrimental effects of thermal exposure. Further research is warranted to optimize the parameters of Ce6-HA and LED irradiation therapy and to explore their potential applications in wound care and regenerative medicine.

**Fig. 7:**
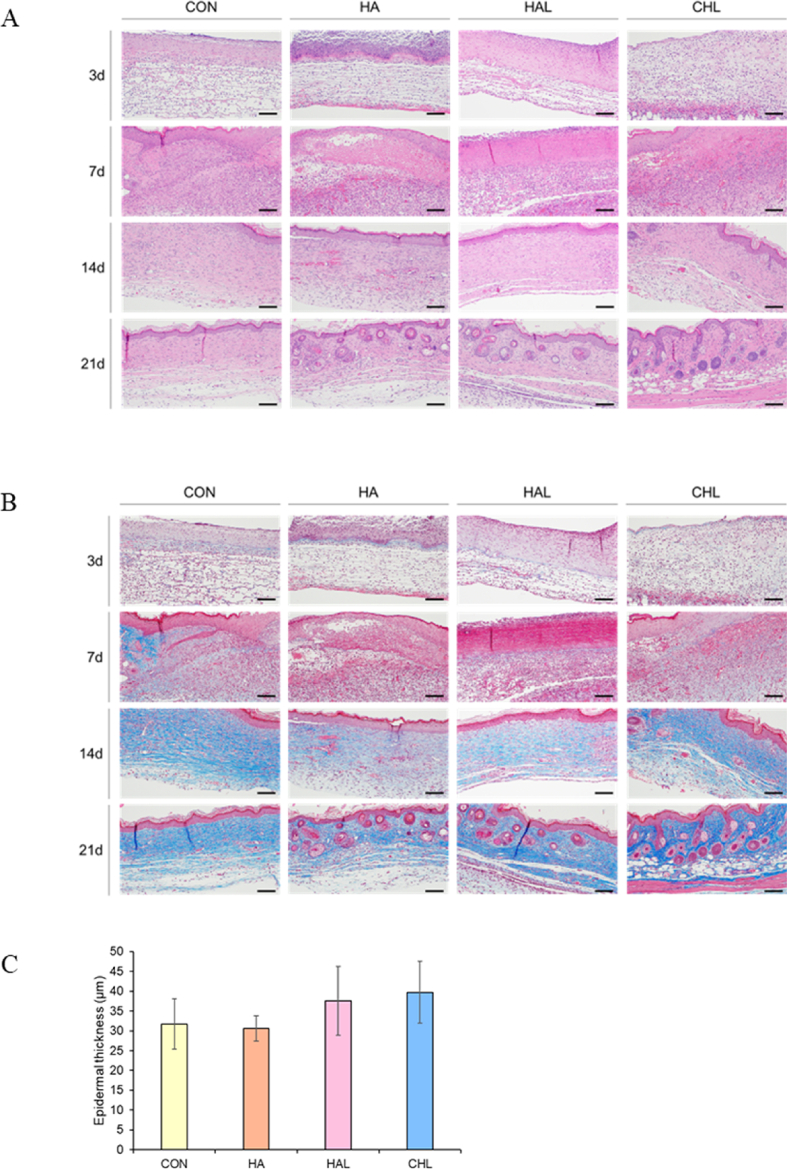
Histological analysis of tissue at various wound healing stages (A) Histological evaluation of wound healing using H&E staining. (B) Collagen deposition analysis using MT staining. (C) Epidermal thickness measurement at post-surgery day 21 (n = 5).

Histological changes in the skin wounds were assessed using H&E and MT staining. On day 3, the wounds exhibited an absence of collagen and irregular epithelial structures in the epidermal layer, with all groups displaying an incomplete epidermis. By day 7, there was an increase in dermal cell infiltration, including mononuclear cells and mast cells, and enhanced angiogenesis, as evidenced by increased epidermal thickness and a more pronounced keratin layer than normal skin. In the control group, an incomplete epidermal structure was observed at the wound center, accompanied by significant cell infiltration into the dermis. In both the HA and HAL groups, partial development of the epidermis was noted, characterized by a thickened appearance with abundant cellular and vascular presence in the dermal layer. Similarly, in the CHL group, the epidermis remained incomplete, but dermal thickening and increased blood vessel formation were observed. By day 14, a normal epidermal structure had been restored. In the CHL group, normal epidermal thickness was observed, along with the presence of hair follicles and sebaceous glands, and collagen accumulation in the dermal layer was evident on MT staining. By day 21, the HA, HAL, and CHL groups showed significant progress in skin structure formation at the wound center compared with the control group. The CHL group demonstrated the closest resemblance to normal skin, indicating the most significant progress in wound healing. These findings suggest that CHL treatment significantly enhances wound healing by promoting the restoration of epidermal structure, stimulating blood vessel growth, and facilitating collagen accumulation in the dermal layer, thereby supporting a more comprehensive process of skin regeneration (Fig. 7A and B). The distinction between the epidermis and dermis was evaluated using H&E and MT staining, which are widely utilized for structural analysis of skin tissue. Based on the analysis of results at day 21, epidermal thickness was measured and compared among experimental groups (Fig. 7C). While the HAL and CHL groups exhibited a slight increase in epidermal thickness, high variability among samples resulted in no statistically significant differences. ROS play a crucial role in mediating the observed effects of the Ce6-HA and LED irradiation treatment on wound healing. ROS influence several key processes in wound repair, including stimulation of fibroblast activity and collagen synthesis, epithelialization, and keratinocyte proliferation [9,12,21,26,55]. ROS can enhance fibroblast proliferation and collagen synthesis, both of which are vital for ECM formation and wound strength [18]. The histological findings showing increased collagen accumulation in the dermal layer of the CHL-treated wounds suggest that ROS played a significant role in upregulating the processes involved in matrix deposition and remodeling. ROS contribute to the re-epithelialization process by promoting keratinocyte proliferation and migration. This is crucial for restoring the skin barrier function [23,24]. The enhanced epidermal thickness and more organized epithelial structures observed in the CHL group indicate that ROS facilitated the rapid coverage of the wound surface by new epithelial cells. Therefore, ROS can positively affect wound healing when present at controlled levels.

### 3.9 Immunostaining analysis for estimating PCNA, CD31, vimentin and CD68

**Fig. 8:**
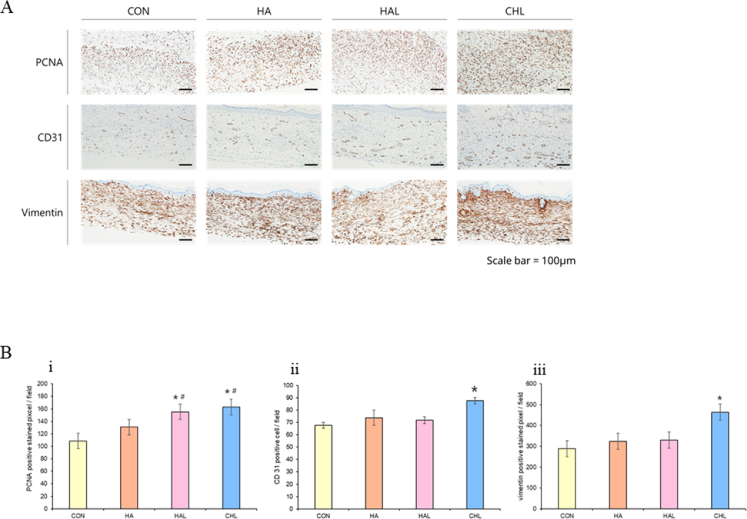
Immunostaining analysis for PCNA, CD31 and vimentin (A) Representative IHC staining for PCNA, CD31, and vimentin. PCNA staining was performed on day 7 samples, while CD31 and vimentin staining were performed on day 14 samples. Scale bar = 100 μm. (B) Quantification of (i) PCNA, (ii) CD31, and (iii) vimentin expression in tissue. Data are presented as mean ± SD. ∗p < 0.05 vs. control, #p < 0.05 vs. HA (n = 3).

PCNA, a protein associated with cell proliferation, was also observed to be more highly expressed in the HAL and CHL groups than in the CON group (Fig. 8B–i). The proliferation of cells observed *in vitro* can be directly correlated with PCNA expression *in vivo*. *In vitro* cell proliferation is mirrored *in vivo* by the upregulation of PCNA in the tissues where cell proliferation is occurring [81].

CD31, a marker of angiogenesis and new blood vessel formation during skin regeneration, was found to be significantly higher in the CHL group than that in the other groups. The increase in the *in vivo* levels of CD31 reflects the physiological relevance of the angiogenic processes observed *in vitro* under the influence of ROS. *In vivo*, the ROS-induced angiogenesis observed *in vitro* translates into increased CD31 expression, as new blood vessels form in response to ROS signaling [82]. This connection underscores the role of ROS as a pivotal regulator of angiogenesis both in cell culture and within living tissues. The controlled induction of ROS in experimental settings, as seen with treatments like Ce6-HA combined with LED irradiation, can enhance angiogenic responses, leading to improved vascularization and tissue repair as indicated by increased CD31 levels *in vivo* (Fig. 8B–ii).

Additionally, vimentin, a protein predominantly expressed in fibroblasts, showed higher expression in the tissue obtained from the CHL group (Fig. 8B–iii). The enhancement of fibroblast proliferation observed *in vitro* directly correlates with the increased expression of vimentin *in vivo* [83]. As fibroblasts proliferate and become more active, they upregulate vimentin, which supports their migration and ECM production [84]. This relationship demonstrates how cellular behaviors studied in controlled *in vitro* environments can translate to meaningful biological markers like vimentin *in vivo*, providing insights into the efficacy of therapeutic strategies aimed at enhancing wound healing and tissue regeneration.

**Fig. 9:**
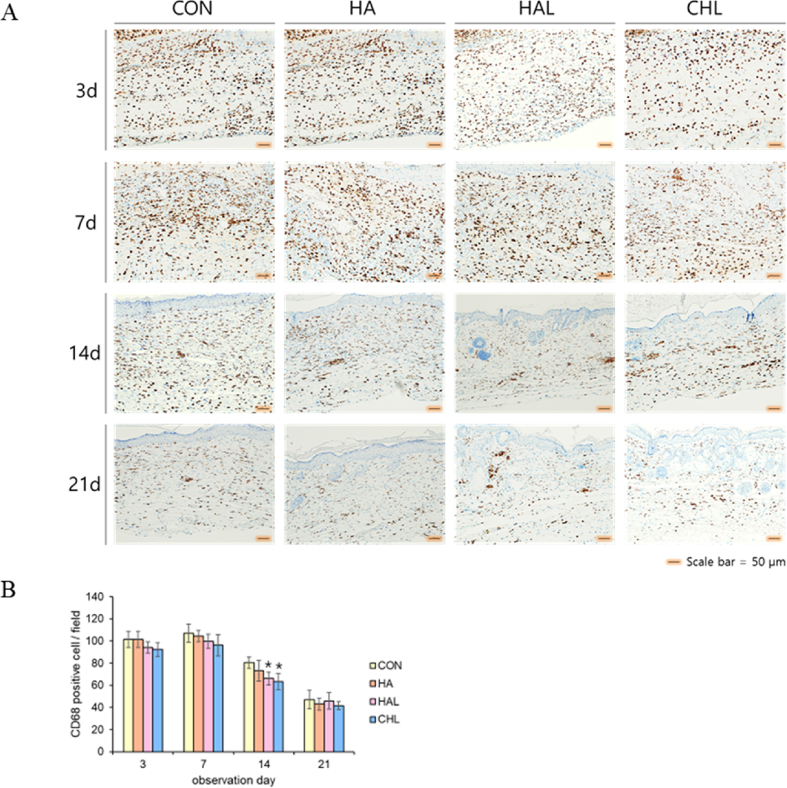
Immunohistochemical analysis of CD68 (A) Representative IHC staining images for CD68 in different groups (CON, HA, HAL, CHL). Scale bar = 50 μm. (B) Quantification of CD68-positive staining in tissue. Data are presented as mean ± SD. ∗p < 0.05 vs. CON (n = 3).

CD68, a macrophage marker, was used to evaluate the inflammatory response of the skin. Following the creation of full-thickness wounds, the innate immune response was immediately activated, leading to macrophage recruitment at the injury site to facilitate the removal of damaged cells and ECM debris [85]. Our results demonstrated that CD68 expression increased across all experimental conditions from Day 3 to Day 7, followed by a gradual decline by Days 14 and 21. Notably, while LED irradiation and ROS generation resulted in slightly lower CD68 expression at Day 14, the overall expression pattern remained consistent among groups. Although BALB/c nude mice are immunodeficient and lack T cells, other immune cells such as macrophages are present and can function. Therefore, when a wound is created, macrophages migrate to the site to suppress inflammation and promote tissue regeneration. The expression of CD68 observed was a result of wound formation, as its levels did not increase due to HA treatment, LED irradiation, or ROS generation. This suggests that the generated ROS remained within a physiologically tolerable range (Fig. 9A and B). ROS play a critical role in immune cell regulation during wound healing, influencing macrophage polarization, neutrophil activation, and T cell responses. Moderate ROS levels promote the transition of macrophages from the pro-inflammatory M1 phenotype to the pro-regenerative M2 phenotype, facilitating tissue repair and extracellular matrix remodeling. However, excessive ROS can sustain inflammation, impair tissue regeneration, and contribute to chronic wound pathology. Similarly, neutrophils rely on ROS for pathogen clearance and inflammatory signaling, but uncontrolled ROS production may lead to excessive tissue damage. Adaptive immune responses are also modulated by ROS, where regulatory T cells (Tregs) benefit from controlled ROS exposure, supporting immune resolution. In contrast, high ROS levels can activate pro-inflammatory pathways, potentially exacerbating chronic inflammation [86].

**Fig. 10:**
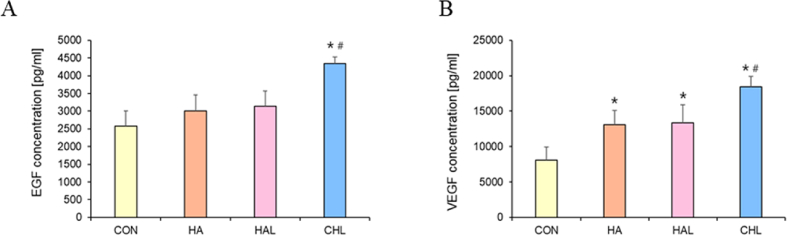
Analysis of cytokines levels in skin tissue (A) EGF and (B) VEGF quantified using ELISA. ∗p < 0.05 vs. con, #p < 0.05 vs. HA (n = 3).

### 3.10 Analysis of growth factors during wound healing

EGF is pivotal in wound healing, as it promotes cellular proliferation, migration, and differentiation. EGF specifically stimulates the formation of granulation tissue, accelerates epithelialization, and facilitates wound closure. Additionally, EGF modulates inflammatory responses and supports angiogenesis, thereby contributing to the overall tissue repair process [87,88]. ROS are known to stimulate the production of EGF, an essential growth factor in wound healing and tissue regeneration. ROS function as signaling molecules that activate key cellular pathways, such as the MAPK pathway, leading to the upregulation of EGF expression [8]. This upregulation occurs as part of the body’s response to injury, where ROS generated at the wound site enhance EGF production, promoting keratinocyte proliferation and migration necessary for re-epithelialization [8,9,22]. Furthermore, ROS can amplify the biological effects of EGF by modulating its receptors and downstream signaling components, thereby reinforcing the regenerative processes [46]. However, while controlled levels of ROS are beneficial, excessive ROS can cause oxidative stress, potentially disrupting these positive effects. Thus, the appropriate levels of ROS are crucial for optimizing EGF-mediated tissue repair.

VEGF is a critical factor in wound healing, primarily by promoting angiogenesis, which ensures sufficient oxygen and nutrient supply to the affected area. VEGF also stimulates the proliferation and migration of endothelial cells, aiding in vascular remodeling and tissue repair. Moreover, VEGF exerts anti-apoptotic effects on endothelial cells, helping them to survive and maintain vascular integrity during the healing process. In summary, VEGF is indispensable for orchestrating angiogenesis and vascular remodeling, both of which are essential for effective wound healing [12,89,90]. ROS are key regulators of VEGF production, which is crucial for angiogenesis [78,79]. ROS stimulate VEGF expression through the activation of signaling pathways, particularly the hypoxia-inducible factor-1 (HIF-1) pathway [91]. Under hypoxic conditions or in the presence of ROS, HIF-1 is stabilized, allowing it to bind to the *VEGF* gene promoter and enhance *VEGF* transcription. Additionally, ROS create a pro-angiogenic environment by upregulating VEGF and other growth factors, thereby promoting endothelial cell proliferation, migration, and new blood vessel formation. ROS also interact with other signaling molecules, such as MAPK and PI3K/AKT pathways, further enhancing VEGF activity and its angiogenic effects [92]. Thus, ROS play a critical role in modulating VEGF-driven angiogenesis, particularly in response to hypoxia and tissue repair requirements.

The findings of our study demonstrate a significant elevation in the levels of EGF and VEGF during the wound healing process in the CHL-treated group (Fig. 10A and B). These elevated levels are consistent with the enhanced wound repair observed in this group, as both EGF and VEGF are critical for promoting cell proliferation, angiogenesis, and tissue regeneration [8,78]. The elevated EGF levels contribute to the proliferation and migration of keratinocytes, which are essential for re-epithelialization and the restoration of the skin barrier [87]. This is particularly important in the early stages of wound healing, where rapid cell proliferation is required to cover the wound bed and initiate tissue repair. In contrast, VEGF plays a crucial role in angiogenesis, stimulating the formation of new blood vessels that supply oxygen and nutrients to the healing tissue [78]. The increased VEGF levels observed in the CHL-treated group likely facilitated improved vascularization of the wound site, which is vital for supporting the metabolic demands of proliferating and regenerating cells. The pivotal role of ROS in stimulating the production of both EGF and VEGF further underscores the importance of ROS in wound healing. By modulating ROS levels through treatments like Ce6-HA combined with LED irradiation, it is possible to enhance the natural healing processes, leading to faster and more efficient wound closure. This aligns with previous reports, which have established a strong correlation between elevated EGF and VEGF levels and improved wound-healing outcomes. These findings suggest that the therapeutic strategy employed in this study could be further optimized and potentially applied in clinical settings to enhance wound repair, particularly in cases where accelerated healing is desired. Further studies could explore the precise mechanisms by which ROS influence the production of these growth factors and investigate the optimal conditions for maximizing their beneficial effects during wound healing.

## 4 Conclusion

Wound healing is a complex, multi-phase process essential for restoring tissue integrity and function after injury. The proliferation of fibroblasts and keratinocytes and the development of new blood vessels (angiogenesis) are critical to this process. In a series of animal studies, ROS-generating HA hydrogels significantly improved wound healing compared with non-ROS-generating HA hydrogels and control groups. The ROS-treated group showed faster and more efficient wound closure, enhanced fibroblast proliferation, increased keratinocyte migration, and more robust angiogenesis. Histopathological and immunohistochemical analyses confirmed these findings, revealing increased expression of key growth factors and signaling molecules involved in wound healing. The release of ROS at optimal levels not only facilitated cellular proliferation and angiogenesis but also activated broader molecular pathways necessary for tissue repair. These results suggest that ROS-triggering HA hydrogels represent a promising therapeutic approach to enhance wound healing, offering faster recovery, reduced scarring, and improved overall outcomes and underscore the potential of this strategy as a focus for future research and development in wound care technologies.

## CRediT authorship contribution statement

**Seung Hee Hong:** Writing – original draft, Methodology, Investigation, Conceptualization. **Ye Jin Park:** Methodology. **Seo In Lee:** Validation. **Ki Chang Nam:** Visualization, Software. **Mi Hee Lee:** Formal analysis. **Jong-Chul Park:** Supervision, Project administration, Conceptualization.

## Ethics approval and consent to participate

The animal study protocols were approved by the Institutional Animal Care and Use Committee (IACUC) of the Yonsei Laboratory Animal Research Center (YLARC; Permit Number: 2023-0136).

## Declaration of competing interest

The authors declare no conflict of interest.

## References

[1] Auger F., Lacroix D., Germain L. (2009). Skin substitutes and wound healing. Skin Pharmacol. Physiol..

[2] Berardesca E., Maibach H. (2003). Ethnic skin: overview of structure and function. J. Am. Acad. Dermatol..

[3] Bhardwaj N., Chouhan D., B Mandal B. (2017). Tissue engineered skin and wound healing: current strategies and future directions. Curr. Pharm. Des..

[4] Cañedo-Dorantes L., Cañedo-Ayala M. (2019). Skin acute wound healing: a comprehensive review. Int. J. Inflamm..

[5] Enoch S., Leaper D.J. (2005). Basic science of wound healing. Surgery (Oxf.).

[6] George Broughton I., Janis J.E., Attinger C.E. (2006). Wound healing: an overview. Plast. Reconstr. Surg..

[7] Gordillo G.M., Sen C.K. (2003). Revisiting the essential role of oxygen in wound healing. Am. J. Surg..

[8] Huo Y., Qiu W.-Y., Pan Q., Yao Y.-F., Xing K., Lou M.F. (2009). Reactive oxygen species (ROS) are essential mediators in epidermal growth factor (EGF)-stimulated corneal epithelial cell proliferation, adhesion, migration, and wound healing. Exp. Eye Res..

[9] Janda J., Nfonsam V., Calienes F., Sligh J.E., Jandova J. (2016). Modulation of ROS levels in fibroblasts by altering mitochondria regulates the process of wound healing. Arch. Dermatol. Res..

[10] Kanta J. (2011). The role of hydrogen peroxide and other reactive oxygen species in wound healing. Acta Med..

[11] Khorsandi K., Hosseinzadeh R., Esfahani H., Zandsalimi K., Shahidi F.K., Abrahamse H. (2022). Accelerating skin regeneration and wound healing by controlled ROS from photodynamic treatment. Inflamm. Regen..

[12] Polaka S., Katare P., Pawar B., Vasdev N., Gupta T., Rajpoot K. (2022). Emerging ROS-modulating technologies for augmentation of the wound healing process. ACS Omega.

[13] Wang G., Yang F., Zhou W., Xiao N., Luo M., Tang Z. (2023). The initiation of oxidative stress and therapeutic strategies in wound healing. Biomed. Pharmacother..

[14] Tracy L.E., Minasian R.A., Caterson E. (2016). Extracellular matrix and dermal fibroblast function in the healing wound. Adv. Wound Care.

[15] Smith P.C., Martínez C., Martínez J., McCulloch C.A. (2019). Role of fibroblast populations in periodontal wound healing and tissue remodeling. Front. Physiol..

[16] Ross R. (1968). The fibroblast and wound repair. Biol. Rev..

[17] Hinz B. (2016). The role of myofibroblasts in wound healing. Curr. Res. Transl. Med..

[18] Darby I.A., Hewitson T.D. (2007). Fibroblast differentiation in wound healing and fibrosis. Int. Rev. Cytol..

[19] Bainbridge P. (2013). Wound healing and the role of fibroblasts. J. Wound Care.

[20] Akita S., Akino K., Hirano A. (2013). Basic fibroblast growth factor in scarless wound healing. Adv. Wound Care.

[21] Gniadecki R. (1998). Regulation of keratinocyte proliferation. Gen. Pharmacol. Vasc. Syst..

[22] Wang Z., Wang Y., Farhangfar F., Zimmer M., Zhang Y. (2012). Enhanced keratinocyte proliferation and migration in co-culture with fibroblasts. PLoS One.

[23] Hsieh S.-T., Lin W.-M. (1999). Modulation of keratinocyte proliferation by skin innervation. J. Invest. Dermatol..

[24] Nita A., Matsumoto A., Tang R., Shiraishi C., Ichihara K., Saito D. (2021). A ubiquitin-like protein encoded by the “noncoding” RNA TINCR promotes keratinocyte proliferation and wound healing. PLoS Genet..

[25] Veith A.P., Henderson K., Spencer A., Sligar A.D., Baker A.B. (2019). Therapeutic strategies for enhancing angiogenesis in wound healing. Adv. Drug Deliv. Rev..

[26] Tonnesen M.G., Feng X., Clark R.A. (2000).

[27] Nie R., Zhang J., Jia Q., Li Y., Tao W., Qin G., Liu X., Tao Y., Zhang Y., Li P. (2024). Structurally oriented carbon dots as ROS nanomodulators for dynamic chronic inflammation and infection elimination. ACS Nano.

[28] Liu H., Ai R., Liu B., He L. (2025). Dual ROS/Glucose-Responsive quercetin-loaded supermolecular hydrogel for diabetic wound healing. Biomacromolecules.

[29] Chen Y., Li Y., Song H., Liu X., Zhang H., Jiang J., Liu H., Zhuo R., Cheng G., Fang J., Xu L., Qi Y., Sun D. (2025). Injectable nanocomposite hydrogel for accelerating diabetic wound healing through inflammatory microenvironment regulation. Int. J. Nanomed..

[30] Abatangelo G., Vindigni V., Avruscio G., Pandis L., Brun P. (2020). Hyaluronic acid: redefining its role. Cells.

[31] Collins M.N., Birkinshaw C. (2008). Physical properties of crosslinked hyaluronic acid hydrogels. J. Mater. Sci. Mater. Med..

[32] Collins M., Birkinshaw C. (2007). Comparison of the effectiveness of four different crosslinking agents with hyaluronic acid hydrogel films for tissue‐culture applications. J. Appl. Polym. Sci..

[33] Burdick J.A., Prestwich G.D. (2011). Hyaluronic acid hydrogels for biomedical applications. Adv. Mater..

[34] Barbucci R., Lamponi S., Borzacchiello A., Ambrosio L., Fini M., Torricelli P. (2002). Hyaluronic acid hydrogel in the treatment of osteoarthritis. Biomaterials.

[35] Bajaj G., Kim M.R., Mohammed S.I., Yeo Y. (2012). Hyaluronic acid-based hydrogel for regional delivery of paclitaxel to intraperitoneal tumors. J. Contr. Release.

[36] Lala D., Rabek J., Ranby B. (1980). The effect of 1, 3-diphenylisobenzofuran on the photo-oxidative degradation of cis-1, 4-polybutadiene. Eur. Polym. J..

[37] Vinodgopal K., Kamat P.V. (1992). Photochemistry on surfaces: photodegradation of 1, 3-diphenylisobenzofuran over metal oxide particles. J. Phys. Chem..

[38] Zhang X.-F., Li X. (2011). The photostability and fluorescence properties of diphenylisobenzofuran. J. Lumin..

[39] Hong S.H., Lee M.H., Go E.J., Park J.-C. (2024). A promising strategy for combating bacterial infections through the use of light-triggered ROS in Ce6-immobilized hydrogels. Regen. Biomater..

[40] Zhang M., Liu X., Mao Y., He Y., Xu J., Zheng F. (2022). Oxygen-generating hydrogels overcome tumor hypoxia to enhance photodynamic/gas synergistic therapy. ACS Appl. Mater. Interfaces.

[41] Addis R., Cruciani S., Santaniello S., Bellu E., Sarais G., Ventura C. (2020). Fibroblast proliferation and migration in wound healing by phytochemicals: evidence for a novel synergic outcome. Int. J. Med. Sci..

[42] Xu H., Zheng Y.-W., Liu Q., Liu L.-P., Luo F.-L., Zhou H.-C. (2018). Reactive oxygen species in skin repair, regeneration, aging, and inflammation. React. Oxyg. Spec. (ROS) Liv. Cell..

[43] Gharee-Kermani M., Pham S. (2001). Role of cytokines and cytokine therapy in wound healing and fibrotic diseases. Curr. Pharm. Des..

[44] Wojtowicz A.M., Oliveira S., Carlson M.W., Zawadzka A., Rousseau C.F., Baksh D. (2014). The importance of both fibroblasts and keratinocytes in a bilayered living cellular construct used in wound healing. Wound Repair Regen..

[45] Werner S., Krieg T., Smola H. (2007). Keratinocyte–fibroblast interactions in wound healing. J. Invest. Dermatol..

[46] Zhang J., Wang X., Vikash V., Ye Q., Wu D., Liu Y. (2016). ROS and ROS‐mediated cellular signaling. Oxid. Med. Cell. Longev..

[47] Görlach A., Bertram K., Hudecova S., Krizanova O. (2015). Calcium and ROS: a mutual interplay. Redox Biol..

[48] Dunn J.D., Alvarez L.A., Zhang X., Soldati T. (2015). Reactive oxygen species and mitochondria: a nexus of cellular homeostasis. Redox Biol..

[49] Nurse P., Masui Y., Hartwell L. (1998). Understanding the cell cycle. Nat. Med..

[50] Kastan M.B., Bartek J. (2004). Cell-cycle checkpoints and cancer. Nature.

[51] Johnson D.G., Walker C.L. (1999). Cyclins and cell cycle checkpoints. Annu. Rev. Pharmacol. Toxicol..

[52] Israels E., Israels L. (2000). The cell cycle. Oncologist.

[53] Barnum K.J., O’Connell M.J. (2014). Cell Cycle Control: Mechanisms and Protocols.

[54] Boraldi F., Lofaro F.D., Bonacorsi S., Mazzilli A., Garcia-Fernandez M., Quaglino D. (2024). The role of fibroblasts in skin homeostasis and repair. Biomedicines.

[55] Day R.M., Suzuki Y.J. (2005). Cell proliferation, reactive oxygen and cellular glutathione. Dose Response.

[56] Yu M., Zheng Y., Sun H.-X., Yu D.-J. (2012). Inhibitory effects of enalaprilat on rat cardiac fibroblast proliferation via ROS/P38MAPK/TGF-β1 signaling pathway. Molecules.

[57] Shi X-m, Xu G-m, Zhang G-j, Liu J-r, Wu Y-m, Gao L-g (2018). Low-temperature plasma promotes fibroblast proliferation in wound healing by ROS-activated NF-κB signaling pathway. Curr. Med. Sci..

[58] Murrell G.A., Francis M.J., Bromley L. (1990). Modulation of fibroblast proliferation by oxygen free radicals. Biochem. J..

[59] Mamalis A., Garcha M., Jagdeo J. (2015). Light emitting diode‐generated blue light modulates fibrosis characteristics: fibroblast proliferation, migration speed, and reactive oxygen species generation. Laser Surg. Med..

[60] Li S., Tabar S.S., Malec V., Eul B.G., Klepetko W., Weissmann N. (2008). NOX4 regulates ROS levels under normoxic and hypoxic conditions, triggers proliferation, and inhibits apoptosis in pulmonary artery adventitial fibroblasts. Antioxidants Redox Signal..

[61] Kim B.-Y., Han M.-J., Chung A.-S. (2001). Effects of reactive oxygen species on proliferation of Chinese hamster lung fibroblast (V79) cells. Free Radic. Biol. Med..

[62] Dunnill C., Patton T., Brennan J., Barrett J., Dryden M., Cooke J. (2017). Reactive oxygen species (ROS) and wound healing: the functional role of ROS and emerging ROS‐modulating technologies for augmentation of the healing process. Int. Wound J..

[63] Liu Y-z, Xu M-y, Dai X-y, Yan L., Li L., Zhu R-z (2021). Pyruvate kinase M2 mediates glycolysis contributes to psoriasis by promoting keratinocyte proliferation. Front. Pharmacol..

[64] Russo B., Brembilla N.C., Chizzolini C. (2020). Interplay between keratinocytes and fibroblasts: a systematic review providing a new angle for understanding skin fibrotic disorders. Front. Immunol..

[65] Wang H., Lei L., Hu J., Li Y. (2020). Oncostatin M upregulates Livin to promote keratinocyte proliferation and survival via ERK and STAT3 signalling pathways. Exp. Physiol..

[66] Zhou P., Feng H., Qin W., Li Q. (2023). KRT17 from skin cells with high glucose stimulation promotes keratinocytes proliferation and migration. Front. Endocrinol..

[67] Zhou X., Chen Y., Cui L., Shi Y., Guo C. (2022). Advances in the pathogenesis of psoriasis: from keratinocyte perspective. Cell Death Dis..

[68] Chong D.L., Trinder S., Labelle M., Rodriguez‐Justo M., Hughes S., Holmes A.M. (2020). Platelet‐derived transforming growth factor‐β1 promotes keratinocyte proliferation in cutaneous wound healing. J. Tissue Eng. Regen. Med..

[69] Barygina V., Becatti M., Lotti T., Moretti S., Taddei N., Fiorillo C. (2019). ROS‐challenged keratinocytes as a new model for oxidative stress‐mediated skin diseases. J. Cell. Biochem..

[70] Raja S.K., Garcia M.S., Isseroff R.R. (2007). Wound re-epithelialization: modulating keratinocyte migration in wound healing. Front. Biosci..

[71] Wei M., He X., Liu N., Deng H. (2024). Role of reactive oxygen species in ultraviolet-induced photodamage of the skin. Cell Div..

[72] Jamil H., Karim N. (2024). Unraveling Mitochondrial reactive oxygen species involvement in psoriasis: the promise of antioxidant therapies. Antioxidants.

[73] Chettouh-Hammas N., Fasani F., Boileau A., Gosset D., Busco G., Grillon C. (2023). Improvement of Antioxidant defences in keratinocytes grown in physioxia: comparison of 2D and 3D models. Oxid. Med. Cell. Longev..

[74] Jomova K., Raptova R., Alomar S., Alwasel S., Nepovimova E., Kuca K., Valko M. (2023). Reactive oxygen species, toxicity oxidative stress, and antioxidants. Chron. Dise. Aging.

[75] Jiang J., Wang K., Chen H., Nice E., Huang C. (2017). Redox regulation in tumor cell epithelial-mesenchymal transition molecular basis and therapeutic strategy. Signal Transduct. Targeted Ther..

[76] Canovas B., Nebreda A. (2021). Diversity and versatility of p38 kinase signaling in the health and disease. Nat. Rev. Mol. Cell Biol..

[77] Benhar M., Engelberg D., Levitzki A. (2002). ROS, stress-activated kinases and stress signaling in cancer.

[78] Zou J., Fei Q., Xiao H., Wang H., Liu K., Liu M. (2019). VEGF‐A promotes angiogenesis after acute myocardial infarction through increasing ROS production and enhancing ER stress‐mediated autophagy. J. Cell. Physiol..

[79] Zhang D.X., Gutterman D.D. (2007). Mitochondrial reactive oxygen species-mediated signaling in endothelial cells. Am. J. Physiol. Heart Circ. Physiol..

[80] Kim Y.-M., Kim S.-J., Tatsunami R., Yamamura H., Fukai T., Ushio-Fukai M. (2017). ROS-induced ROS release orchestrated by Nox4, Nox2, and mitochondria in VEGF signaling and angiogenesis. Am. J. Physiol. Cell Physiol..

[81] Zhang B., Liu P., Zhou Y., Chen Z., He Y., Mo M. (2019). Dihydroartemisinin attenuates renal fibrosis through regulation of fibroblast proliferation and differentiation. Life Sci..

[82] Fukai T., Ushio-Fukai M. (2020). Cross-talk between NADPH oxidase and mitochondria: role in ROS signaling and angiogenesis. Cells.

[83] Cheng F., Shen Y., Mohanasundaram P., Lindström M., Ivaska J., Ny T. (2016). Vimentin coordinates fibroblast proliferation and keratinocyte differentiation in wound healing via TGF-β–Slug signaling. Proc. Natl. Acad. Sci..

[84] Coelho-Rato L.S., Parvanian S., Modi M.K., Eriksson J.E. (2024). Vimentin at the core of wound healing. Trends Cell Biol..

[85] Kim H., Son K., Son Y., Kin Y., Lee K., Lee S., Suh J., Lee J. (2022). A comparative immunohistochemical study of wound healing after dental diode laser treatment in the rat oral mucosa. Bioengineering.

[86] Robinson H., Jarrett P., Vedhara K., Tarlton J., Whiting C., Law M., Broadbent E. (2022). The effect of expressive writing on wound healing: immunohistochemistry analysis of skin tissue two weeks after punch biopsy wounding. J. Psychosom. Res..

[87] Shakhakarmi K., Seo J.-E., Lamichhane S., Thapa C., Lee S. (2023). EGF, a veteran of wound healing: highlights on its mode of action, clinical applications with focus on wound treatment, and recent drug delivery strategies. Arch Pharm. Res. (Seoul).

[88] Li Y., Leng Q., Pang X., Shi H., Liu Y., Xiao S. (2021). Therapeutic effects of EGF-modified curcumin/chitosan nano-spray on wound healing. Regen. Biomater..

[89] Truong A.-T.N., Kowal-Vern A., Latenser B.A., Wiley D.E., Walter R.J. (2005). Comparison of dermal substitutes in wound healing utilizing a nude mouse model. J. Burn. Wound..

[90] Bryan N., Ahswin H., Smart N., Bayon Y., Wohlert S., Hunt J.A. (2012). Reactive oxygen species (ROS)–a family of fate deciding molecules pivotal in constructive inflammation and wound healing. Eur. Cell. Mater..

[91] Cheng J., Yang H.-L., Gu C.-J., Liu Y.-K., Shao J., Zhu R. (2019). Melatonin restricts the viability and angiogenesis of vascular endothelial cells by suppressing HIF-1α/ROS/VEGF. Int. J. Mol. Med..

[92] Hutchings G., Kruszyna Ł., Nawrocki M.J., Strauss E., Bryl R., Spaczyńska J. (2021). Molecular mechanisms associated with ROS-dependent angiogenesis in lower extremity artery disease. Antioxidants.

