# Supplementary material for: Photodynamically tunable ROS-generating hydrogels for accelerated tissue regeneration
Source: Bioact Mater. 2025 Jul 8;51:977–92. doi: 10.1016/j.bioactmat.2025.05.006 (PMC12273516; doi:10.1016/j.bioactmat.2025.05.006)
Supplement: Multimedia component 1 [file mmc1.docx]

**Supplementary data**

S1. *In vivo* preliminary studies to determine the optimal ROS conditions for wound healing.


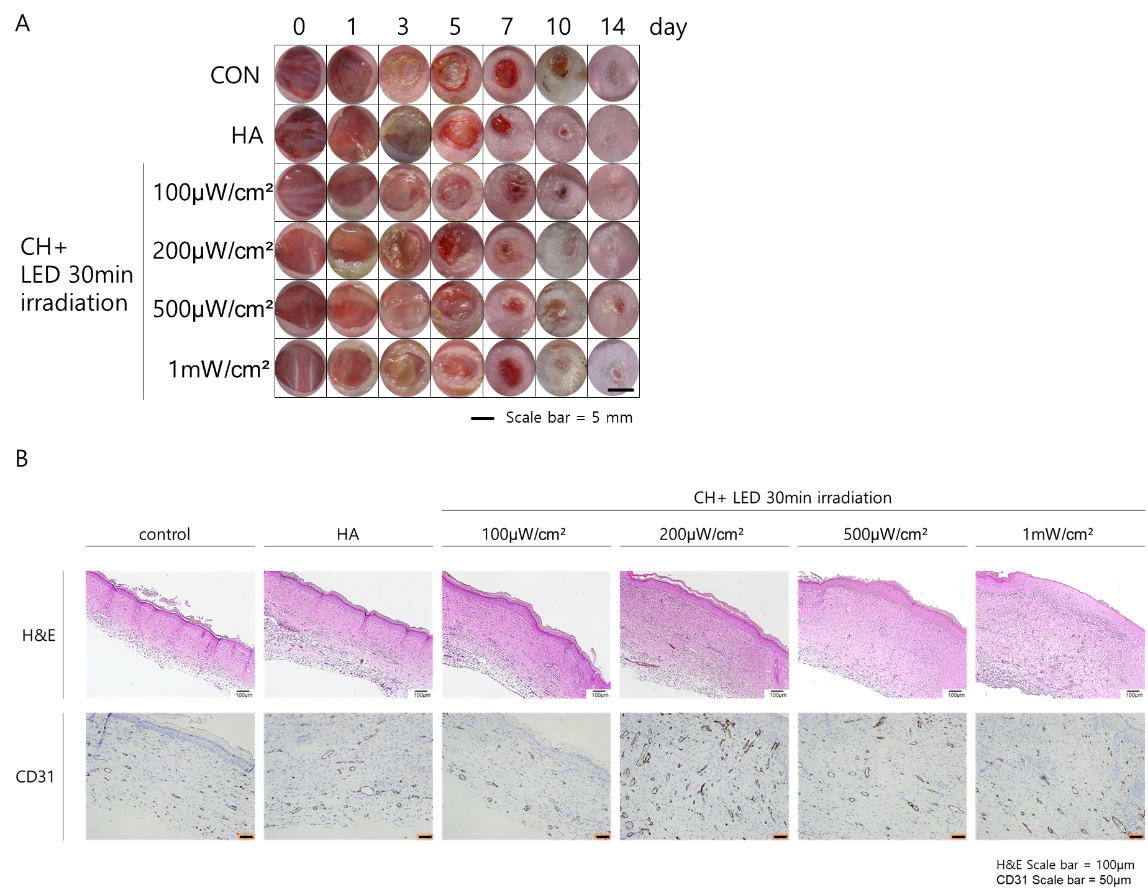


To determine the optimal LED irradiation intensity for *in vivo* experiments, preliminary studies were conducted to assess ROS generation and its effects on wound healing under different LED intensities. Various light intensities (100, 200, 500 µW/cm², and 1 mW/cm²) were applied for 30 min to Ce6-HA-treated wounds. Wound healing response was evaluated through macroscopic imaging and CD31 IHC staining to assess angiogenesis. The results demonstrated that *in vivo*, 200 µW/cm² for 30 min yielded the most effective wound healing outcome.

The rationale for selecting different LED intensities for *in vitro* and *in vivo* experiments lies in the differences in light penetration, ROS generation efficiency, and tissue interactions. *In vitro*, cells are directly exposed to uniform light without interference from biological structures, and the culture environment maintains a stable oxygen supply, allowing for efficient ROS production at a lower intensity (100 µW/cm²). However, *in vivo*, light penetration is affected by tissue absorption, scattering, and diffusion, leading to reduced ROS generation compared to direct irradiation in cell culture. To achieve a comparable level of ROS generation and biological effects, a higher intensity (200 µW/cm²) was required *in vivo*. This adjustment compensates for the attenuation of light within the tissue and ensures sufficient ROS-mediated signaling for optimal wound healing.

Thus, based on these findings, 100 µW/cm² was selected for *in vitro* experiments, while 200 µW/cm² was chosen for *in vivo* experiments to achieve consistent ROS-mediated effects under different experimental conditions.
